# Urinary Tract Infection among Post-renal Transplant Patients in the Department of Nephrology of a Tertiary Care Centre: A Descriptive Cross-sectional Study

**DOI:** 10.31729/jnma.7496

**Published:** 2022-06-30

**Authors:** Bikash Khatri, Suresh Maharjan, Jagdish Lamsal, Bijay Khatri, Dibya Singh Shah

**Affiliations:** 1 Department of Nephrology, National Academy of Medical Sciences, Kathmandu, Nepal; 2 Department of Nephrology, Shahid Dharmabhakta National Transplant Centre, Dudhpati, Bhaktapur, Nepal; 3 Academic and Research Department, Hospital for Children Eye ENT and Rehabilitation Service, Lokanthali, Bhaktapur, Nepal; 4 Department of Nephrology, Institute of Medicine, Tribhuvan University Teaching Hospital, Maharajgunj, Kathmandu, Nepal

**Keywords:** *Escherichia coli*, *hypertension*, *kidney*, *transplants*

## Abstract

**Introduction:**

Urinary tract infection is the most common infection among renal transplant recipients and increases the risk of hospitalization or even death. The study aimed to find the prevalence of urinary tract infection among post-renal transplant patients in the Department of Nephrology of a tertiary care centre.

**Methods:**

This is a descriptive cross-sectional study which was conducted among 217 post-renal transplant patients at the Department of Nephrology of a tertiary care centre from 1^st^ November, 2017 to 31^st^ October, 2018. The study was approved by the Institutional Review Committee (Reference number: 245(6-11-E)2074-75). Convenience sampling was used. The data were entered in Microsoft Excel 2011 and analyzed using the Statistical Package for the Social Sciences version 20.0. Point estimate at 95% Confidence Interval was calculated along with frequency and proportion for binary data and mean with standard deviation for continuous data.

**Results:**

Among 217 patients, urinary tract infection was seen in 27 (12.44%) (8.05-16.83 at 95% Confidence Interval). One (3.70%) patient had the infection within three months of transplant, and 17 (62.96%) had infection after more than a year of transplant.

**Conclusions:**

The prevalence of urinary tract infection among kidney transplant recipients in our study was lower than previous studies done in similar settings.

## INTRODUCTION

Kidney transplantation improves the quality of life and overall survival with a lower cost than dialysis and is, therefore, the treatment of choice for EndStage Renal Disease (ESRD) patients. Advances in immunosuppressive therapy have decreased acute rejection rates and dramatically improved graft survival to more than 60% after 10 years.^1,2^ Nevertheless, the immunosuppressive regimens required to avoid rejection increase the risk of infection.^3,4^

Urinary Tract Infection (UTI) is the most common complication following kidney transplantation, which could result in bacteremia, acute T cell-mediated rejection, impaired allograft function, and allograft loss, with an increased risk of hospitalization and death.^5,9^

This study aimed to find the prevalence of UTI among renal transplant recipients in a tertiary care centre.

## METHODS

This is a descriptive cross-sectional study conducted at Tribhuvan University Teaching Hospital (TUTH), Kathmandu, Nepal. The study was approved by the Institutional Review Committee of the Institute of Medicine, Maharajgunj (Reference number: 245(6-11-E)2074-75). The patients who underwent renal transplantation in the Department of Nephrology, TUTH, were the study population. Only the transplant recipients who came to the same department from 1^st^ November, 2017 to 31^st^ October, 2018 for follow-up were included in the study. The patients who underwent renal transplants from other centers or outside Nepal were excluded from the study. Convenience sampling was used in this study. The sample size was calculated using the following formula:


$$\begin{array}{ll} \text{n} & ={\text{Z}}^{2}\times \frac{\text{p}\times \text{q}}{{\text{e}}^{\text{2}}}\\ & =1.96^{2}\times \frac{0.5\times 0.5}{0.07^{2}}\\ & =196 \end{array}$$

Where,

n = minimum required sample size
Z = 1.96 at 95% Confidence Interval (CI)
p = prevalence taken as 50% for maximum sample size calculation
q = 1-p
e = margin of error, 7%

The calculated sample size was 196. However we have taken 217 transplant recipients. UTI has been defined as the presence of bacteriuria on laboratory reports and receipt of one or more courses of antibiotics. This definition of UTI therefore included all cases of acute simple cystitis, transplant pyelonephritis and asymptomatic bacteriuria if treated with antibiotics, which was done if there was an unexplained rise in serum creatinine or deemed clinically necessary.^10^ A semi-structured questionnaire was developed from literature review and expert advice as a study proforma. The patients were interviewed by their consulting nephrologist, and their OPD records were reviewed. All the patients were invited voluntarily after a clear explanation of the study objectives. Written informed consent was obtained from all the patients and the patients' parties.

The data were entered in Microsoft Excel 2011 and analyzed using the Statistical Package for the Social Sciences (SPSS) version 20.0. Point estimate at 95% CI was calculated along with frequency and percentages for binary data and mean with standard deviation for continuous data.

## RESULTS

Out of 217 transplant recipients coming to the OPDs of the Department of Nephrology, there was at least one episode of UTI among 27 (12.44%) (8.05-16.83 at 95% Confidence Interval) patients. During the study period, those 27 patients presented with 57 episodes of UTI.

The mean age of patients was 41.6±13.4 years, and 17 (62.96%) were male. The most common native kidney disease was hypertension in 11 (40.74%) patients. Fifteen (55.56%) had received a kidney from biologically related donors. The level of Human Leukocyte Antigen (HLA) mismatch was 0 in 1 recipient (3.70%), and 18 (66.67%) had 4 to 6 mismatches. Induction with rabbit anti-thymocyte globulin was given in 25 (92.59%) of the patients. Nearly two-thirds, 17 (66.6%) of the patients had received transplants more than a year ago (Table 1).

**Table 1:** General characteristics of Urinary Tract Infection patients (n = 27).

Characteristics	n (%)
**Gender**	
Male	17 (62.96)
Female	10 (37.04)
**Native kidney diseases**	
Hypertension	11 (40.74)
Chronic glomerulonephritis	8 (29.63)
Diabetes mellitus	4 (14.81)
Obstructive uropathy	1 (3.70)
Chronic interstitial nephritis	1 (3.70)
Undetermined aetiology	2 (7.41)
**Donor**	
Blood-related	15 (55.56)
Non-blood related	12 (44.44)
**HLA mismatch**	
0	1 (3.70)
1 to 3	8 (29.63)
4 to 6	18 (66.67)
**Use of induction immunosuppression**	
Rabbit Anti-thymocyte Globulin (rATG)	25 (92.59)
Daclizumab	1 (3.70)
Simulect	1 (3.70)
**Duration of transplant**	
Less than 3 months	1 (3.70)
3-6 months	3 (11.11)
6-12 months	6 (22.22)
More than 1 year	17 (62.96)

In patients with UTI, 24 (88.89%) had positive urine cultures. The most common organism among 24 positive urine cultures was *Escherichia coli* in 13 (54.17%) (Table 2).

**Table 2:** Bacterial isolates from renal transplant recipients (n = 24).

Bacteria	n (%)
*Escherichia coli*	13 (54.17)
*Klebsiella species*	5 (20.83)
*Enterococcus faecalis*	2 (8.33)
*Pseudomonas species*	2 (8.33)
*Acinetobacter species*	1 (4.17)
*Streptococcus species*	1 (4.17)

Among 57 episodes of UTI recorded during the study period, 17 (29.82%) episodes required hospital admissions.

## DISCUSSION

Twenty-seven patients had at least one episode of UTI during the study period. There were 57 episodes of UTI among 27 patients. *Escherichia coli* was the most common bacterial species isolated from the urine culture. TUTH was selected purposely as this tertiary center has regularly been the pioneer institution in renal transplantation and post-transplant care in Nepal since 2008.^11^

The prevalence of UTIs in our study was 12.44%. At the inception of the transplantation program in the same institute, the prevalence of UTI was 38.7%, which is higher than our present study.^12^ This might be due to better surgical skills gained over the years, better immunosuppressive medication use, and lesser instrumentations which is a prime factor causing UTIs in transplant patients. The incidence of UTI is reported to have fallen over the past years; nonetheless, it is desirable to reduce as it improves the quality of life among the patients.^13^

In our study, the most common organism isolated from infected patients was *Escherichia coli* (54.2%). A similar observation was seen in various studies, including another study done in similar setting where the incidence was 36%.^12^ Another study in Australia showed *Escherichia coli* again to be the commonest organism causing UTIs in transplant recipients.^10^ In a setting similar to ours also found *Escherichia coli* to cause 51% of UTIs in renal transplant recipients.^14^ The main causative organism of UTI is similar to those causing UTIs in the general population, like *Escherichia coli.* However, resistant pathogens such as extended-spectrum lactamase-producing *Klebsiella*, vancomycin-resistant *Enterococcus* species, and *Pseudomonas aeruginosa* are all significant pathogens a study which was similar to our findings.^15^ Similarly, the majority of the causative microorganisms of UTI episodes were *Escherichia coli* (56.1%) and *Enterococcus species* (24.6%) in a study.^16^ These organisms need to be considered in the prophylaxis regimen of UTI to prevent recurrent infections.

UTI was common in the male patients in our study (63%). In other studies, the percentage of infection was more common in females after renal transplantation.^10^ This discrepancy might be due to gender-biased renal transplant recipients in our part of the world, where up to 71% of the transplant recipients are male.^11^

Our study demonstrates that UTI is more common in patients whose renal transplant duration exceeded one year. This might be due to more patients following up in OPD over the study duration of a year who had undergone transplants years before exceeding those who underwent a transplant in the study year. A study demonstrated that more episodes of UTI occurred in patients who had undergone transplants more than six months ago in the same institute.^12^ However, another study showed that 77% of UTIs occur in the first two months after a renal transplant.^14^ Similarly, studies have reported UTIs in 74% of patients during the first month after kidney transplantation.^17^ This is important because UTI may have a long-term impact on graft outcome.^9^

Diabetic nephropathy has also been widely reported to be associated with an increased risk of UTI.^18^ This was not observed in our study. This could be due to the small number of diabetic patients in the study group. Other causes of ESRD were not found to be a significant cause of increasing the risk of post-transplant urinary tract infections. There was no difference between the induction agents used or the immunosuppressive medicines used.

A study has shown that both graft and patient survival in their study was significantly lower in renal transplant patients who had infections than those in control without infection, confirming a negative impact of infection on the outcome of the kidney transplant patient. The outcome of patients with recurrent infections seemed to be worse than that of those who had a single episode of infection.^19^ Hence, the risk factors associated with UTIs should be prioritized in transplant recipients as at least one episode of UTI among transplant recipients is associated with declining renal function.^20^

This study was limited to patients coming for follow-up at TUTH only and could not include other transplant recipients following at other care centers. Besides, the study was descriptive cross-sectional and focused only on the prevalence and episodes of UTIs. There is a paucity in the etiology of the infection among the transplant recipients in Nepal, and it is advisable to conduct the study.

## CONCLUSIONS

The prevalence of UTI was found to be lower than in similar studies conducted in similar settings. However, the physicians should be advising transplant patients on possible infection of UTI and its consequences, including death. More research needs to be conducted to find the factors associated with UTIs among transplant recipients and ways to minimize the infection.
